# Marine Antimicrobial Peptides: Nature Provides Templates for the Design of Novel Compounds against Pathogenic Bacteria

**DOI:** 10.3390/ijms17050785

**Published:** 2016-05-21

**Authors:** Annarita Falanga, Lucia Lombardi, Gianluigi Franci, Mariateresa Vitiello, Maria Rosaria Iovene, Giancarlo Morelli, Massimiliano Galdiero, Stefania Galdiero

**Affiliations:** 1 Department of Pharmacy, CIRPEB-University of Naples “Federico II”, Via Mezzocannone 16, 80134 Napoli, Italy; annarita.falanga@unina.it (A.F.); giancarlo.morelli@unina.it (G.M.); 2 Department of Experimental Medicine, II University of Naples, Via De Crecchio 7, 80138 Napoli, Italy; lucia.lombardi@unina2.it (L.L.); gianluigi.franci@yahoo.it (G.F.); mteresa.vitiello@unina2.it (M.V.); MariaRosaria.IOVENE@unina2.it (M.R.I.); massimiliano.galdiero@unina2.it (M.G.); 3 John Felice Rome Center, Loyola University Chicago, Via Massimi 114, 00136 Roma, Italy

**Keywords:** antimicrobial peptide, membrane bilayer, microbial resistance, marine AMPs

## Abstract

The discovery of antibiotics for the treatment of bacterial infections brought the idea that bacteria would no longer endanger human health. However, bacterial diseases still represent a worldwide treat. The ability of microorganisms to develop resistance, together with the indiscriminate use of antibiotics, is mainly responsible for this situation; thus, resistance has compelled the scientific community to search for novel therapeutics. In this scenario, antimicrobial peptides (AMPs) provide a promising strategy against a wide array of pathogenic microorganisms, being able to act directly as antimicrobial agents but also being important regulators of the innate immune system. This review is an attempt to explore marine AMPs as a rich source of molecules with antimicrobial activity. In fact, the sea is poorly explored in terms of AMPs, but it represents a resource with plentiful antibacterial agents performing their role in a harsh environment. For the application of AMPs in the medical field limitations correlated to their peptide nature, their inactivation by environmental pH, presence of salts, proteases, or other components have to be solved. Thus, these peptides may act as templates for the design of more potent and less toxic compounds.

## 1. Introduction

The indiscriminate worldwide overuse and misuse of antibiotics has led to high rates of microbial resistance [1,2], posing new challenges to human health. Hence, we are facing a worldwide re-emergence of infectious diseases and a rapid increase in pathogenic multidrug-resistant (MDR) bacteria, resistant to commercially available antibiotics, threatening the world with a return to the pre-antibiotic era. The search of novel molecules with antibacterial activity that could overcome the resistance phenomenon is a priority. Therefore, naturally-occurring, cationic antimicrobial peptides (AMPs) are considered the new hope and attract attention of scientists as suitable templates for the development of alternatives to conventional antibiotics. AMPs have been isolated from a variety of organisms [3] and have been shown to play a key role in building their defense strategies, being part of the humoral natural defense against infections [4]. The number of AMPs effective against pathogenic bacteria continues to grow. They are relatively small peptides (<60 amino acids) with a broad-spectrum of activity against microorganisms (Gram-positive and Gram-negative bacteria, fungi, viruses, parasites) [5] and a low likelihood of developing resistance [6,7]. Moreover, AMPs may play multifunctional roles which extend far beyond their ability to function as antibiotics. As a matter of fact, some of these peptides have anticancer activity, stimulate the immune system by favoring cytokine release, chemotaxis, antigen presentation, angiogenesis, inflammatory responses, and adaptive immune induction [8].

Much effort is devoted to attaining novel antimicrobial compounds with broad spectrum activity against a wide spectrum of pathogenic microorganisms and recent research is focused on the development of modified compounds to obtain more selective and efficient drugs.

In contrast to most antibiotics, which usually target specific steps in bacterial growth and replication, AMPs’ antimicrobial activity is correlated to membrane disruption and osmotic lysis of microbes and/or to acting on intracellular targets of microbes inhibiting the synthesis of proteins, nucleic acids, and the cell wall. The mechanism used by AMPs to kill specific bacteria exhibits notable differences from peptide to peptide and specificity for particular AMP-bacteria coupling; moreover, a clear relationship between AMP structures and killing mechanisms has not yet arisen. It has also been proposed that molecules able to disrupt the membrane bilayer are a promising alternative to antibiotics since they present the additional advantage of being active against dormant bacteria with slow or no growth. In fact, dormant bacteria can survive high concentrations of antibiotics and need extensive treatment for efficacy [9,10].

As for the mechanism inducing the damage of the membrane and/or internalization, AMPs essentially exploit major differences in the composition of bacterial *versus* eukaryotic membranes. Selectivity is in fact correlated to the difference between membrane compositions and characteristics of host and pathogen cells. Differences, such as the absence of cholesterol, greater presence of anionic lipids, and a stronger inward directed electric field are key for the specificity. Moreover, it is more difficult for bacteria to alter these features compared to the more circumscribed molecular targets of conventional antibiotics and development of resistance is unlikely because it would require a change in the bacterial membrane.

**Figure 1:**
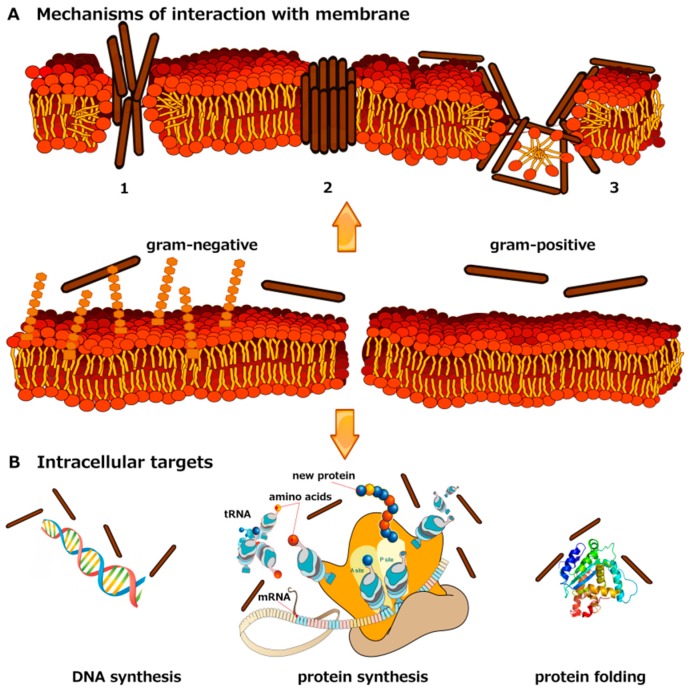
Mechanisms inducing the damage of the membrane and/or internalization (**panel A**); The main proposed mode of action are: carpet model (1); barrel stave model (2) and toroidal-pore model (3). Alternative mechanisms of antibacterial activity involving intracellular targets (**panel B**). Some AMPs act on intracellular targets inhibiting cell-wall synthesis, nucleic acid binding and synthesis, protein production, and enzyme activity.

Biophysical studies have provided models for the mechanism of membrane damage; the main proposed mode of action are the carpet model, barrel stave model, and toroidal-pore model [11]. In all proposed models, the initial interaction between the peptide and the bacterial membrane is electrostatic and involves the positively-charged residues in the peptides and the negatively-charged moieties on the surface of the bacterial membrane. The main features of the three models (Figure 1) are reported below.

According to the carpet model, peptides accumulate in a parallel fashion on the lipid membrane surface forming a carpet-like structure. Following the initial electrostatic interaction between AMPs and the phospholipids, the peptide reaches a threshold concentration and inserts into the membrane, breaking the lipid structure and causing cell lysis in a detergent-like manner involving a large-scale micellization of the bilayer. This mode of action has been proposed for peptides, like dermaseptin and cecropins, with positively-charged amino acids distributed along the peptide sequence which do not cause hemolysis because of their weak interaction with zwitterionic membranes [12].

The barrel-stave model is typical of α-helical peptides with spatially-separated, and distinctly-hydrophobic and -hydrophilic regions; moreover, the net charge of these amphipathic peptides is close to neutral. Amphipathic α-helices insert into the hydrophobic core of the membrane establishing interactions with lipid polar head groups using their hydrophilic portion and interactions with the hydrophobic chains using its hydrophobic portion. As a result, transmembrane pores are formed. According to this model, peptides, such as alamethicin, bind to the membrane, recognize each other and oligomerize, the oligomer inserts into the hydrophobic core of the membrane, forming a transmembrane pore inserted perpendicular to the bilayer surface [12].

The toroidal-pore model is typical of AMPs, such as magainins, protegrins, and melittin. This model differs from the barrel-stave model in a way that peptides are always associated with lipid head groups even when they are perpendicularly inserted into the highly-curved lipid surfaces [12].

Recently, Marrink *et al.* [13], using molecular dynamic simulations, proposed a “chaotic pore” model, involving a continuously changing situation characterized by a localized permeabilization caused by a time varying number of peptide and lipid molecules.

In conclusion, both cationic amino acid residues and hydrophobic residues play key roles in the interaction of peptides with phospholipid bilayer and subsequent membrane perturbation.

The absence of a clear correlation between structure and function, further supports the idea that interfacial activity determines the ability of a peptide to permeabilize membranes [14]. This is key, also, to distinguish between antibacterial peptides able to damage the membrane bilayer and cell-penetrating peptides able to cross the bilayer without damages [4,15,16,17]. In fact, some cell-penetrating peptides are also antibacterial and the switch is correlated to their concentration [4,15].

Alternatively, some AMPs act on intracellular targets inhibiting cell-wall synthesis, nucleic acid binding and synthesis, protein production, and enzyme activity.

As part of the ongoing global effort to discover novel antimicrobials to treat infections caused by resistant pathogenic organisms, many laboratories are now devoted to the discovery of novel antimicrobial compounds against antibiotic resistant bacteria and putative antibacterial compounds are directly derived from the plant and animal kingdoms. The marine environment is extremely hydrophilic and contains not only a wide range of microorganisms but also a high salt content. In this review, we will describe a selection of AMPs derived from marine organisms and microorganisms (sponges, algae, bacteria, fungi, and fish). Marine AMPs have been reviewed thoroughly in other reviews [18,19,20]. However, in the present paper, we will briefly describe several classes of marine AMPs and their modifications.

## 2. Marine-Derived AMPs

Marine environment constitutes more than 70% of the Earth and is associated with such a chemical diversity that it represents an enormous resource for the discovery of potential therapeutic agents. The marine environment is constituted by approximately 10^6^ bacteria/mL and 10^9^ virus/mL of seawater and, thus, represents a rich source of pathogens. Marine organisms live in close proximity with pathogenic microbes; thus, in order to survive in such a harsh environment they need to have a robust and effective immune system, and AMPs constitute the first line of defense against invading microbes. Marine AMPs have been shown to be structurally different from their analogues derived by terrestrial species and often present novel structures [21,22]. Moreover, AMPs’ antimicrobial activity is based on their initial electrostatic interaction with the negatively-charged surface of the bacteria; thus, free ions produced by the high salt concentrations in the surrounding medium, typical of some illnesses, could efficiently decrease interaction and antimicrobial activity. Marine AMPs have evolved to adapt to the high salt concentration in sea water and probably this has been achieved by the substitution of lysines with arginines. Therefore, marine organisms are a promising source of novel bioactive substances for the development of therapeutics.

Recently, numerous bioactive compounds with appealing pharmaceutical activities have been derived from the marine environment. Certainly, marine biodiversity is inimitable and, in fact, between 50% and 80% of all lifeforms are present only in oceans. Of the 33 known animal phyla, 32 are present in aquatic environment, among which 21 are exclusively marine.

In this review, we will focus on AMPs isolated from the main marine organisms and microorganisms of interest: sponges, algae, fish, *etc.*, which represent the richest source of pharmacologically-active molecules (Table 1).

Marine AMPs differ in length, isoelectric point (pI), secondary structure, hydrophobicity, and amphipathicity, and in their antimicrobial activities. Notwithstanding the number and diversity of marine AMPs, there are some key structural arrangements shared by most of them allowing the broad classifications into the following categories based on their structural features and biochemical characteristics [23], namely: (a) linear α-helical peptides; (b) linear or helical peptides with abundance of one amino acid (proline, tryptophan, histidine or glycine rich peptides); (c) peptides forming hairpin-like β-sheet or α-helical/β-sheet mixed structures stabilized by intramolecular disulfide bonding; and (d) cyclic peptides.

a. *Linear α-helical peptides*. These peptides are short sequences with spatially-separated hydrophobic and hydrophilic regions in a linear structure which are believed to attain a helical conformation upon interaction with the membrane. Among the α-helical peptides are styelin, clavanin A, piscidin, pleurocidins, mixinidin, and hedistin [24,25].

Several studies have shown that piscidin is able to form pore and cause disruption of bacterial membranes also at high salt concentrations [26]; the C-terminal α-helix is important for the antibacterial activity and cell selectivity of the peptide [27].

Pleurocidin is a 25 amino acid peptide, whose potent antibacterial activity has been attributed to its N and C terminal hydrophobic regions [28,29]. Pleurocidin can actively kill bacteria up to 625 mM NaCl which is approximately the concentration of salt in the marine environment [29]. Epinecidin (Epi)-1 is a 21 amino acid peptide that was first identified in a fish species *Epinephelus coioides* [30]. It is structurally similar to the peptide pleurocidin and it is active against infection with Gram-negative bacteria, including *P. aeruginosa*, *Vibrio vulnificus*, and *Riemerella anatipestifer* [30].

Styelins A and B [31,32] are two α-helical phenylalanine-rich antimicrobial peptides effective against a panel of Gram-negative and Gram-positive bacteria with minimal inhibitory concentrations <1.5 μg/mL (<0.5 μM), even in the presence of 100 mM NaCl.

Papillosin and halocyntin [33] are two cationic AMPs with strong activity against Gram-positive and Gram-negative bacteria. However, papillosin presents an activity eight times higher than halocyntin [33].

Dicynthaurin, [34] is composed of two 30-residue cynthaurin monomers containing only four lysines and no arginines or histidines. Although many antimicrobial peptides with α-helical conformations are known, dicynthaurin is unusual because it contains an unpaired cysteine and forms covalent homodimers. Moreover, dicynthaurin has broad-spectrum activity against both Gram-positive and Gram-negative bacteria. Although dicynthaurin is a marine AMP, it has been suggested that it has an intracellular target (e.g., within a phagosome) because its antimicrobial activity is optimal at NaCl concentrations below 100 mM. Myxinidin is an antimicrobial peptide derived from the epidermal mucus extract of the hagfish *Myxine glutinosa L.* [35,36,37]. It shows activity against a broad range of bacteria and its activity is retained at concentrations up to 0.3 M NaCl.

Parasin I is a 19-residue AMP isolated from the skin of the catfish *Parasilurus asotus* [38,39], and it shows potent antimicrobial activities against a broad spectrum of gram-positive and Gram-negative bacteria.

Clavanins A, B, C, D and E, contain 23 amino acid residues and are C-terminally amidated [40]. Clavanin A was demonstrated to effectively kill *Micrococcus flavus* and permeabilize its cytoplasmic membrane at micromolar concentrations [41].

Hedistin is an antimicrobial peptide derived from the coelomocytes of *Nereis diversicolor* [42] which showed potent microbicidal activities against a broad range of bacteria and contains bromotryptophan residues.

b. *Linear or helical peptides with abundance of one amino acid.* These peptides present an over-representation of one amino acid. Although the role played by amino acids such as proline, glycine, tryptophan, and histidine in these peptides has not been clarified, it is widely accepted that these amino acid residues are essential for their antimicrobial activity [23].

Arasin contains several prolines and arginines at N-terminus, and four cysteine residues forming two disulfide bonds at the C-terminus [43,44]. Hyastatin, derived from hemocytes of *Hyas araneus*, comprises three distinct domains: the N-terminal region rich in glycine residues, a short domain rich in arginine and proline residues and a C-terminal region with cysteine residues which is key for activity [45].

Callinectin is a cationic AMP derived from the blue crab *Callinectes sapidus* [46,47]. Callinectin is a 32 amino acid, proline- and arginine-rich AMP with four cysteine residues. This peptide exists as three isomers which vary in the functional group on the tryptophan residue. The most prevalent isomer has a hydroxyl-N-formylkynurenine group. Callinectin has significant amino acid sequence similarity to arasins. However, arasin 1 has a free C-terminus and presents no modifications on its tryptophan residue. Callinectin and arasins have a proline and arginine rich N-terminus and a cysteine motif at the C-terminus. A similar cysteine motif is present in other AMPs including mammalian protegrins, tachyplesins, polyphemeusins, androctonin, and gomesin from arthropods, because of its high similarity with arasin 1, we included this peptide in the linear/helical peptides with abundance of one or more amino acids; but this sequence could also fit the group of peptides with disulfide bonds.

Three chrysophsin peptides have been identified in red sea bream; they are cationic α-helical peptides rich in histidine residues [48,49]. In addition to displaying secondary amphipathicity, due to the separation of hydrophobic and charged residues upon adoption of the secondary helical structure, they also present a considerable change in hydrophobicity between the N and C terminus, which is correlated to the presence of the RRRH motif at their C-terminus. At intermediate peptide concentration, they are aligned parallel to the membrane surface destabilizing the lipid acyl chains, in agreement with models where permeabilization is correlated to the transient membrane disruption [50]. Moreover, the C-terminus RRRH sequence has a large effect on the insertion into membranes with different compositions and is fundamental for pore formation.

Astacidin 2 is a 14 amino acid residue proline/arginine-rich antibacterial peptide isolated from hemocytes of the freshwater crayfish, *Pacifastacus leniusculus* [51,52]. Astacidin 2 has a broad range of antibacterial activity against both Gram-positive and Gram-negative bacteria.

Collagencin is an AMP derived from fish collagen hydrolysate. The peptide is rich in proline and glycine residues and was shown to adopt a β-sheet structure under hydrophobic conditions. The peptide showed a broad spectrum antibacterial activity with *S. aureu*s being the most sensitive [53].

c. *Peptides forming hairpin-like β*-*sheet or α-helical/β-sheet mixed structures stabilized by intramolecular disulphide bonding*. This group of peptides is cysteine-rich, contains β-sheet and disulfide bonds. These peptides display a pattern with three or four disulfide bonds which stabilize the mixed α-helical/β-sheet scaffold. Several authors have proposed that the presence of the fourth disulfide bond confers additional stability and compactness in high-salt concentrations typical of seawater environment.

The main examples of this group are defensins. Differently from mammals, where three types of defensins have been identified based on their structure (namely α, β and θ defensins) [54,55,56], fish defensins are solely β-defensins with the conserved six-cysteine motif. Fish β-defensins have been shown to be active against both Gram-negative and Gram-positive bacteria, although with rather moderate activity [57,58,59].

Aurelin is a 40 residue peptide derived from the jelly fish *Aurelia aurita*, which is very similar to defensins with three disulfide bonds [60,61]. Arenicins are 21 residue peptides highly active against *P. aeruginosa* and *S. aureus*, which was proved to have as main target the bacterial cell membrane [62,63,64]. Mylitin, another marine AMP in this category, has been shown to lose its antimicrobial activity when its cysteine-stabilized αβ structure is destroyed [65]. Myticin A was isolated from mussels *Mytilus galloprovincialis* [66]. Myticin A is a cyclic peptide containing eight cysteines with marked activity against Gram-positive bacteria and is much less active against Gram-negative bacteria and fungi.

Hepcidins are cysteine-rich peptides with antimicrobial activity against both Gram-positive and Gram-negative bacteria. Fish hepcidins were first identified in hydrid striped bass and since then in many other fish species [67]. The general structure is characterized by the presence from four to eight cysteine residues (from two to four disulfide bonds).

Damicornin is a 40 residue AMP derived from a scleractinian coral of *Pocillora damicornis* [68]. Damicornin is cationic, C-terminally amidated, and characterized by the presence of six cysteine residues joined by three intramolecular disulfide bridges. Damicornin is active against Gram-positive bacteria and the fungus *Fusarium oxysporum*, but presents little activity against Gram-negative bacteria.

Tachyplesin I is an antimicrobial cationic peptide of 17 residues found in the hemocyte debris of horseshoe crab *Tachypleus tridentatus* [69,70]. Tachyplesin I assumes a fairly rigid conformation constrained by two disulfide bridges and adopts a conformation consisting of an anti-parallel β-sheet connected by a β-turn [71]. In this conformation, it assumes an amphipathic structure which is presumed to be closely associated with its bactericidal activity. Even at low concentrations, tachyplesin I has strong activity against both Gram-positive and Gram-negative bacteria.

Poliphemusins are 18 amino acid residue peptides containing four cysteine residues, similarly to tachyplesin I. They show strong activity against both Gram-negative and Gram-positive bacteria, but also against fungi [71].

Penaeidins are a family of peptides isolated from the shrimp *Penaeus annamei* [72] displaying antimicrobial activity predominantly against Gram-positive bacteria. The overall structure of this class of peptides is unique in that it consists of a NH_2_-terminal domain rich in proline residues and a cysteine-rich COOH-terminal region. The COOH-terminal domain contains six cysteines engaged in the formation of three intramolecular disulfide bridges. Penaeidins contain the same number of cysteines as the arthropod and mammalian defensins.

Strongylocins are two novel antibacterial peptides from coelomocyte extracts of the green sea urchin, *Strongylocentrotus droebachiensis* [73]. The two peptides (named strongylocins 1 and 2) are cationic, cysteine-rich, defensin-like peptides; interestingly, they do not show similarity to other known AMPs concerning the cysteine distribution pattern but have potent activities against Gram-negative and Gram-positive bacteria.

Crustins are cationic cysteine-rich antibacterial peptides present in diverse crustaceans, including crabs, shrimps, and crayfish. The first crustin was isolated from the shore crab *Carcinus maenas* [74]; all reported crustin belong to three types (Type I, II, and III). Type I crustins contain a single acidic protein (WAP) domain at the carboxyl terminus and eight cysteine residues; the cysteines fold to form four disulfide bonds and create a tightly packed structure [75,76,77]. Type II crustins contain a glycine rich profile, a cysteine rich profile and a WAP domain [78]. Type III crustins lack the cysteine rich domain and glycine rich domain and contain a proline-arginine rich region together with the WAP domain [79].

Ls-Stylicin1 is derived from the penaeid shrimp, *Litopenaeus stylirostris*. The negatively-charged peptide contains a proline-rich N-terminal region and a C-terminal region with 13 cysteine residues [80]. Its poor antibacterial activity with MICs in the range of 40–80 μM, support the view that the typical direct membrane disruption is unlikely. Ls-Stylicin1 was proved to interact with LPS present in the cell wall of Gram-negative bacteria, and more particularly LPS from *V. penaeicidae* and *E. coli*. Recently, a novel stylicin from Kuruma shrimp *Marsupenaeus japonicas* (Mi-sty) has been identified [81].

d. *Cyclic peptides.* Discodermin A is a 14 amino acid polypeptide isolated from a sea sponge, *Discodermia kiiensis*, with significant antimicrobial activity against Gram-positive and Gram-negative bacteria and fungi [82]. Discodermin A contains two t-Leu residues and several d-amino acids. Other cyclic peptides have been isolated from marine environment, but most of them show antimicotic activities and often antibacterial activity has not been assayed.

This classification of marine AMPs based on their scaffold has several shortcomings and may not account for their mechanism of action. Despite several peptides fall within a similar structural class, their modes of action may vary greatly. For example, tachyplesis, polyphemusin, and arecin, despite adopting a classic β-hairpin fold stabilized by either 1 or 2 disulfide bonds, have been proved to have a broadly different mechanism of action. Tachyplesin and polyphemusin translocate across the membrane without significant disruption of the lipid bilayers, probably acting intracellularly. Arenins disrupt the cell membrane through the formation of higher order oligomers which are responsible of pore formation. Hystatin and paenedins were here classified into two different groups. Paenedins display a high content of Pro/Gly/Arg in their N-terminal extended domain and an α-helical C-terminal domain stabilized by the presence of three disulfide bonds. This scaffold is rather unusual because α-helical peptides display their antimicrobial activity without the need of further stabilization. The N-terminal domain is believed to be involved in the initial interaction with the membrane. Hystatin possesses an N-terminal domain similar to paenedins, rich in Pro/Gly.

Another important feature is the fact that major conformational changes occur on contact with membranes of microorganisms. For example, the α-helix is often fundamental for the antibacterial activity. The interaction with lipids may induce conformational changes and often helix formation which might be involved in penetration. The modification of the α-helical content in the structure of peptides has been carried out by several groups but this change is not always beneficial [83]; in fact, the antimicrobial potency may be related to the inducibility of a helical conformation in a membrane mimicking environment rather than to the intrinsic helical stability. The amphipathic conformation independently from the secondary structure also plays a key role, allowing the peptide to insert the hydrophobic face into the bilayer.

## 3. Challenges in the Use of AMPs as Drugs

Many reports describe the potential role of AMPs as antimicrobial agents and the possibility to exploit them to solve the problem of resistance. In particular, although there is a wealth of information on their activities *in vitro*, there are considerable challenges for their clinical application. These include doubts on the ability to achieve high antimicrobial activities under physiological salt, pH, and serum conditions; the rapid degradation by proteases; the poor oral availability; the difficult transportation across cell membranes; the non-selective receptor binding; the lack of information about potential toxicities *in vivo*; and the challenging multistep preparations and consequently high costs associated with their production [84].

Moreover, AMPs often lack efficacy compared to conventional antibiotics. One possible strategy that has been exploited to improve the antimicrobial efficacy and reduce the toxicity is the combined use of two or more molecules. For example, colistin and bacteriocin have been used together to attain a synergistic effect and overcome some defects. Colistin is a polypeptide antibiotic which was withdrawn because of its toxicity, but when used in combination with bacteriocidin it was effective at lower concentrations.

Rational design has gained great importance and represents a major revolution in the area of development of AMPs which are more active, less cytotoxic and possible to produce on an industrial scale. Bioinformatic and biophysical studies aimed at identification of physicochemical features such as appropriate hydrophobicity, charge, amphipathic structural arrangement, and amphipathicity have acquired a key role. Natural AMPs may serve as templates for the design of new antibacterial agents. Thus, peptidomimetics, which structurally mimic the key binding elements of the native peptide and retain the ability to interact with the biological target and produce the same biological effect, offer a strategy to overcome the issues correlated to the use of peptides in clinical therapeutics [84]. In particular, to develop improved AMPs by structural modification, it is essential to understand the structure of native AMPs and to focus on regions responsible for activity. AMPs can be optimized to enhance their effectiveness and stability through modification of their primary sequences in order to obtain good templates for the development of therapeutic agents. Studies of AMP activity have often included the systematic change of amino acids or other chemical modifications which allow the obtainment of higher activities such as: chemical modification of terminal ends of peptides [85], development of analogues containing unnatural amino acids [86], shortening of the native sequence, modifications of their amphipathic character, cyclization. The appropriate balance of hydrophobicity, amphipathicity, and positive charge plays a pivotal role for the enhancement of their therapeutic potential. The reduction in the hydrophobicity determines a reduction of mammalian cell interactions while favoring the targeting of bacterial cell membranes, as long as the peptide has sufficient positive charge.

Surface immobilization of AMPs also represents an attractive contact-killing technique which could further enhance AMP stability, broad range of action, and the low likelihood for the development of microbial resistance, reducing leaching, proteolysis, and cytotoxicity [87].

Several authors have described modification of native sequences to dissect the mechanism of antibacterial activity modulating changes in hydrophobicity, length, and net charge of the peptides.

## 4. Chemical Modification of Marine AMPs

Synthetic and modified AMPs derived from marine peptides can sustain physiological salt concentration and protease activity.

The collection of post-translational modifications exploited by marine AMPs may help in the design of AMPs with enhanced stability and efficacy, for therapeutic applications in humans. In particular, the high salinity (up to 600 mM) of the marine environment makes it likely that marine AMPs naturally possess greater salt resistance than those derived from other sources, which may allow them to keep their biological activities in relatively high-salt environments, such as in saliva, gastrointestinal fluid, serum, or other body fluids.

Marine AMPs undergo various post-translational modifications, which play a key role in the survival of marine organisms; most of them are required to induce proper folding into structural scaffolds that are necessary for the interactions with target bacterial surfaces and their membranes and to provide the necessary stability [88].

Post-translational modifications include: disulfide bonds, bromination, chlorination, C-terminal amidation, high content of specific amino acids (such as phenylalanine, arginines), modification of single amino acids (such as 3-methylisoleucine), presence of fatty acid linked to the peptide sequence, and presence of d-amino acids. Some are specific to marine AMPs and other modifications are shared with terrestrial AMPs.

Bromination is observed in cathelicidins and protects the peptide from proteases in marine environment, as also evidenced by the absence of bromination of cathelicidins derived from terrestrial mammals [89].

Callinectin occurs in three different form which vary according to the modification present on the tryptophan residue [47]. Modified tryptophans have been found in AMPs from a number of aquatic animals. The most frequent modification is bromination (bromotryptophan). Strongylocins contain a bromotryptophan residue in their sequence which makes them less susceptible to protease digestion, which is a key feature of AMPs with biotechnological potential [73]. The presence of bromotryptophan is associated with increased resistance to proteolysis. A hydroxylated tryptophan is present in MGD-2, an arthropod defensin from *Mytilus galloprovincialis* [90] and in piscidin 4 [91]. The presence of the modified tryptophan is in some cases needed for full expression of antimicrobial activity; in fact, MGD-2 without tryptophan hydroxylation presents reduced activity against certain Gram-negative bacteria [73].

Styelin contains unusual amino acids such as dihydroxyarginine, dihydroxylysine, 6-bromotryptophan, and 3,4-dihydroxyphenylalanine which are important for the antimicrobial activity at high salt concentrations [92]. Polydiscamide A contains a 3-methylisoleucine. Halicylindronides show a N-terminus blocked by a formyl group and a further lactonized C-terminal threonine. This modification provides proteolytic resistance and is sometimes found as an alternative to C-terminal amidation. As stated, the interaction of cationic peptides with anionic membranes represent the initial step of the antibacterial mechanism, thus AMP activity may be compromised by the presence of high salt concentrations. Pleurocidin can actively kill bacteria up to 625 mM NaCl [29]; another AMP derived from the marine invertebrate *Ciona intestinalis* can resist salt concentrations up to 450 mM [93].

Marine AMPs have evolved to adapt to the high salt concentration in sea water and this has probably been achieved by the substitution of lysines with arginines. Understanding the chemical reason that supports this salt independent activity could aid in the design of novel AMPs that could address pathogens under a wide range of normal and abnormal salt concentrations. In fact, human beta defensin-1 (hBD-1) is unable to inhibit *P. aeruginosa* due to a 120 mM concentration of NaCl in the lungs of cystic fibrosis patients [94]; while β-defensin-3 (hBD-3) shows antibacterial activity also at high salt concentrations [54,55]. As a matter of fact, hBD-3 contains a C-terminal domain rich in arginine residues which has been demonstrated to be involved in the activity at high ionic strength through the synthesis of chimeric peptides obtained from hBD-1 and hBD-3 [54,55,95]. Therefore, the understanding of the general rules underlining the ability of an AMP to inhibit microorganisms under physiological salt concentrations (120–150 mM) is a significant aspect in the success of an AMP under *in vivo* studies.

Rational modification of the amino acid sequence of mixinidin further supported the key role played by an appropriate balance of hydrophobicity, amphipathicity, and positive charge in α-helical peptides for the enhancement of their therapeutic potential. The highest activity was obtained for the WMR peptide, which contains an extra tryptophan residue and three arginines (His3, Asp4, and Pro11 were substituted with arginines). The simultaneous substitutions of residues present in positions 3, 4, and 11 with Arg and the addition of the Trp at the N-terminus result in a significant increase in activity [36,37].

Mai *et al.* [96] designed a small, target-specific, salt-resistant AMP that selectively killed *S. mutans*, combining the targeting domain of the *S. mutans* (ComC signaling peptide) to an active portion of pleurocidin which was highly effective against all *S. mutans* strains. The obtained molecule was capable of retaining antibacterial activity in physiological or even higher concentrations of salt compared to its native peptide. Interestingly, the molecule was also able to exert its antibacterial activity at low pH, (pH 5.5 to 6.0) though with a 20% to 25% lower activity than that at pH 7.5.

Cardoso *et al.* [97] designed a novel polyalanine-rich cationic peptide, named Pa-MAP 1.9, which was rationally designed based on the HPLC-8 peptide derived from the polar fish *Pleuronectes americanus*. They proved that the peptide is a valuable candidate for the treatment of gram-negative bacterial infections especially in their biofilm state; moreover, it is not chemolytic and cytotoxic against mammalian cells. The interaction with the bacterial membrane is favored by the adoption of an α-helical conformation which helps the orientation and insertion into lipid bilayers mimicking the bacterial membrane.

## 5. Conclusions

Nature is a rich and still-undisclosed source of bioactive molecules for developing novel drugs against serious bacterial pathogens and to contrast the rapid emergence of multi drug resistance. This review article provides a brief description of marine AMP scaffolds amenable to the development of therapeutic candidates. In particular, marine microorganisms, which usually experience extreme and stressful environments, are becoming a rich source of templates for the design of novel AMPs which could be developed into effective drugs for human and veterinary medicine. The structure of marine AMPs may be exploited to achieve novel sequences with greater activity and stability in high ionic strength environments because marine organisms are constantly under an enormous microbial challenge from the ocean environment, which is also continuously altered by industrialization and transportation.

The need to produce novel molecules able to reduce the possibility of developing resistance has enhanced the interest toward molecules which target the cell membrane for which no resistance has been reported [4,98,99,100,101,102,103]. Thus, the difficulties in accomplishing resistance against AMPs by the pathogens make them potential substitutes for antibiotics and marine AMPs present us with a vast resource of biomolecules for assessing novel therapeutics as antimicrobials. Moreover, use of combinatorial therapeutics should be promising in the future.

Marine peptides have also a high potential for the nutraceutical and medicinal industry and some of them are already on the market or in different phases of the clinical and preclinical pipeline [22].

Treatment of MRSA-infected mice with epenecidin-1 [30] allowed mice to survive by considerably decreasing the bacterial counts with further evidence of wound closure and angiogenesis enhancement [104]. Epinecidin-1 is a promising candidate for topical application against vaginal or skin infections and to have a synergistic effect with commercial cleaning solutions. Moreover, its activity was not influenced by low pH or storing at room temperature and at 4 °C for up to 14 days [105].

Interestingly, chrysophsin-1 has been used to create antimicrobial surfaces capable of killing around 82% of *E. coli* bacteria [106].

Moreover, bacterial infections are rarely unique and occur as polymicrobial communities (biofilms). The anti-biofilm activity against *Candida albicans*, *P. aeruginosa*, and *Bacillus pumilus* of a small peptide isolated from the marine *Bacillus liqueniformis D1* has been reported [107]. This peptide is also able to inhibit preformed biofilms at concentrations equivalent to MIC for planktonic cells, a feature which plays essential key role in fighting chronic established infections.

Bioactive compounds from the marine environment possess properties of biomedical importance, which make them attractive templates for rational design strategies of new drugs and pharmaceuticals with huge biotechnological and pharmaceutical potential. New technologies, close collaboration between researchers of various fields and increasing economic support will be key to the development of marine peptides as novel therapeutics.

## Figures and Tables

**Table 1:** Selection of some peptide from different classes.

Structure	Example of Marine AMPs	Organism	Microorganisms: Bacteria	Antibacterial Activity	Length	Ref.
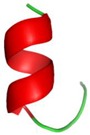 Linear α-helical	Clavanins A, B, C, D and E	Solitary tunicate: *Styela clava*	Gram-negative: *E. coli*, *S. typhimurium*, *P. aeruginosa*	0.4–5 μM (MIC)	23	[40]
			Gram-positive: *S. aureus*, *M. flavus*			
	Dicynthaurin	Solitary tunicate: *Halocynthia aurantium*	Gram-negative: *E. coli, P. aeruginosa*	140 μg/mL (MIC)	60	[34]
			Gram-positive: *M. luteus*, *L. monocytogenes*, *S. aureus*			
	Halocyntin	Ascidian: *Halocynthia papillosa*	Gram-negative: *E. coli, P. aeruginosa*, *S. typhimurium*, *K. pneumoniae*	0.75–100 μM (MBC)	26	[33]
			Gram-positive: *M. luteus*, *B. megaterium*, *A. viridans*, *S. aureus*, *E. facecolis*			
	Hedistin	Annelid: *Nereis diversicolor*	Gram-negative: *V. alginolyticus*	0.4–1.6 μM (MIC)	22	[42]
			Gram positive: *M. luteus*, *M. nishinomiyaensis*, *S. aureus*			
	Myxinidin	Hagfish: *Myxine glutinosa*	Gram-negative: *E. coli*, *P. aeruginosa*, *S. Typhimurium*, *K. Pneumoniae*	5–30 μM (MIC)	12	[35,36,37]
			Gram-positive: *S. aureus*			
	Papillosin	Ascidian: *Halocynthia papillosa*	Gram-negative: *E. coli*	0.25–1 μM (MBC)	34	[33]
			Gram-positive: *M. luteus*, *S. aureus*			
	Parasin 1	Catfish: *Parasilurus asotus*	Gram-negative: *E. coli*, *P. putida*, *S. enteriotidis*	2–4 μg/mL (MIC)	19	[38,39]
			Gram-positive: *B. subtilis*, *S. aureus*, *S. mutans*			
	Pleurocidin	Winter flounder: *Pleuronectes americanus*	Gram-negative: *E. coli*, *C. aquatilis*, *S. typhimurium*	1.1–17.7 μM (MIC)	25	[28,29]
			Gram-positve: *B. subtilis*, *S. aureus*			
	Piscidins 1, 2, 3, 4 and 5	FISH: *Nila tilapia*	Gram-negative: *E. coli*, *P. vulgaris*, *V. vulnificus*, *P. aeruginosa*	0.6–23.6 μg/mL (MIC)	21–44	[26]
			Gram-positive: *S. aureus*, *B. cereus*, *E. faecalis*			
	Styelins A, B, C, D and E	Solitary tunicate: *Styela clava*	Gram-negative: *E. coli*, *S. typhimurium*, *P. aeruginosa*	1–3 μg/mL (MIC)	32	[31,32]
			Gram-positive: *L. monocytogenes*, *E. faecium*, *S. aureus*			
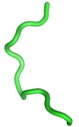 Linear or helical with abundance of one amino acid	Arasin 1 (proline and arginine rich)	Spider crab: *Hyas araneus*	Gram-negative: *L. anguillarum*, *E. coli*	0.8–12.5 μM (MIC)	37	[43,44]
			Gram-positive: *C. glutamicum*			
	Astacidin 1 and 2 (proline and arginine rich)	Crayfish: *P. leniusculus*	Gram-negative: *S. flexneri*, *P. vulgaris*, *E. coli*, *P. aeruginosa*	0.5–15 μM (MIC)	14–16	[51,52]
			Gram-positive: *S. aureus*, *B. megaterium*, *B. subtilis*, *M. luteus*			
	Callinectin (proline, arginine and cysteine rich)	Blue crab: *Callinectes sapidus*	Gram-negative: *E. coli*	1.44 μM (MBC)	32	[46,47]
	Chrysophsin 1, 2 and 3 (histidine rich)	Red Sea Bream: *Chrysophrys major*	Gram-negative: *E. coli*	4–16 μg/mL (MIC)	20–25	[48,49,50]
			Gram-positive: *B. subtilis*, *S. mutans*			
	Hyastatin (glycine rich)	Spider crab: *Hyas araneus*	Gram-negative: *E. coli*	0.4–12.5 μM (MIC)	131	[45]
			Gram-positive: *C. glutamicus*			
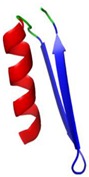 Forming hairpin-like β-sheet or α-helical-β-sheet mixed structures with disulfide bonds	Arenicins 1, 2 and 3	Polychaete: *Arenicola marina*	Gram-negative: *E. coli*, *P. aeruginosa*	2–8 μM (MIC)	21	[62,63,64]
			Gram-positive: *L. monocytogenes*, *S. aureus*, *S. epidermidis*			
	Aurelin	Jelly fish: *Aurelia aurita*	Gram-negative: *E. coli*	7.6–22.6 μg/mL (MIC)	40	[60,61]
			Gram-positive: *L. monocytogenes*			
	Crustins types I, II and III	Crab: *Carcinus maenas; Portunus trituberculatus*	Gram-negative: *P. aeruginosa*, *V. alginolyticus*	1.5–49.6 μM (MIC)	77–95	[75,76]
			Gram-positive: *M. luteus*, *S. aureus*			
	Damicornin	Coral: *Pocillopora damicornis*	Gram-positive: *M. luteus*, *B. megaterium*, *S. aureus*, *B. stationis*, *M. maritypicum*	1.25–20 μM (MIC)	40	[68]
	Hepcidins	Seabream: *Sparus aurata*	Gram-negative: *E. coli*, *A. hydrophila*, *V. prahaemloyticus*	6–24 μM (MIC)	26	[67]
			Gram-positive: *B. subtilis*, *M. luteus*, *S. aureus*			
	MCdef	Manila clams: *Ruditapes philippinarum*	Gram-negative: *V. logei*, *V. salmonicida*	1.25–20 μM (MIC)	44	[59]
			Gram-positive: *S. aureus*, *S. iniae*			
	Myticins A, B and C	Mussel: *Mytilus galloprovincialis*	Gram-negative: *E. coli*, *S. typhimurium*, *P. aeruginosa*	2.25–20 μM (MBC)	40	[66]
			Gram-positive: *M. luteus*, *B. megaterium*, *E. viridans*			
	Mylitin	Mussel: *Mytilus galloprovincialis*	Gram-negative: *V. splendidus*, *V. anguillarum*, *E. coli*	125 μM–2 mM (MIC)	34	[65]
			Gram-positive: *M. lysodeikticus*			
	Penaeidins 1, 2 and 3	shrimp: Penaeus vannamei	Gram-positive: *B. megaterium*, *A. viridans*	0.3–2.5 μM (MIC)	50–60	[72]
	Poliphemusin	American horseshoe crab: *Limulus polyphemus*	Gram negative: *E. coli*	0.25 μM (MIC)	18	[71,108]
	Strongylocins 1 and 2	green sea urchin: *Strongylocentrotus droebachiensis*	Gram-negative: *E. coli*, *L. anguillarum*	1.3–5 μM (MIC)	83–90	[73]
			Gram-positive: *S. aureus*, *C. glutamicum*			
	Tachyplesins I, II and III	Horseshoe Crab: *Tachypleus tridentatus*	Gram-negative: *E. coli*, *S. thyphimurium*	0.6–6.3 μg/mL (MIC)	17–18	[69,70,71]
			Gram-positive: *S. aureus*			
Cyclic peptides	Discodermin A	Marine sponge: *Discodermia kiiensis*	Gram-negative: *P. mirabilis*, *P. morganii*	1.56–12.5 μg/mL (MIC)	14	[82]
			Gram-positve: *B. subtilis*			
